# Imputation of Ordinal Outcomes: A Comparison of Approaches in Traumatic Brain Injury

**DOI:** 10.1089/neu.2019.6858

**Published:** 2021-01-29

**Authors:** Kevin Kunzmann, Lorenz Wernisch, Sylvia Richardson, Ewout W. Steyerberg, Hester Lingsma, Ari Ercole, Andrew I.R. Maas, David Menon, Lindsay Wilson

**Affiliations:** ^1^ MRC Biostatistics Unit, Cambridge Institute of Public Health, University of Cambridge, Cambridge, United Kingdom.; ^2^ Department of Public Health, Erasmus MC, Rotterdam, the Netherlands.; ^3^ Department of Biomedical Data Sciences, LUMC, Leiden, the Netherlands.; ^4^ Center for Medical Decision Sciences, Erasmus MC, Rotterdam, the Netherlands.; ^5^ Division of Anaesthesia, University of Cambridge, Addenbrooke's Hospital, Cambridge, United Kingdom.; ^6^ Department of Neurosurgery, Antwerp University Hospital and University of Antwerp, Edegem, Belgium.; ^7^ Division of Psychology, University of Stirling, Stirling, United Kingdom.

**Keywords:** GOSe, imputation, missing data, traumatic brain injury

## Abstract

Loss to follow-up and missing outcomes data are important issues for longitudinal observational studies and clinical trials in traumatic brain injury. One popular solution to missing 6-month outcomes has been to use the last observation carried forward (LOCF). The purpose of the current study was to compare the performance of model-based single-imputation methods with that of the LOCF approach. We hypothesized that model-based methods would perform better as they potentially make better use of available outcome data. The Collaborative European NeuroTrauma Effectiveness Research in Traumatic Brain Injury (CENTER-TBI) study (*n* = 4509) included longitudinal outcome collection at 2 weeks, 3 months, 6 months, and 12 months post-injury; a total of 8185 Glasgow Outcome Scale extended (GOSe) observations were included in the database. We compared single imputation of 6-month outcomes using LOCF, a multiple imputation (MI) panel imputation, a mixed-effect model, a Gaussian process regression, and a multi-state model. Model performance was assessed via cross-validation on the subset of individuals with a valid GOSe value within 180 ± 14 days post-injury (*n* = 1083). All models were fit on the entire available data after removing the 180 ± 14 days post-injury observations from the respective test fold. The LOCF method showed lower accuracy (i.e., poorer agreement between imputed and observed values) than model-based methods of imputation, and showed a strong negative bias (i.e., it imputed lower than observed outcomes). Accuracy and bias for the model-based approaches were similar to one another, with the multi-state model having the best overall performance. All methods of imputation showed variation across different outcome categories, with better performance for more frequent outcomes. We conclude that model-based methods of single imputation have substantial performance advantages over LOCF, in addition to providing more complete outcome data.

## Introduction

Assessments of global functional outcome such as the Glasgow Outcome Scale (GOS) and the Glasgow Outcome Scale extended (GOSe) are used across the full spectrum of recovery, and have popularity as end-points in traumatic brain injury.^1,2^ However, missing outcome data is a common problem in TBI research, and for longitudinal studies, completion rates at 6 months can be <70%.^3^ This is important, because complete-case analyses may introduce bias and at least reduce power.^4^

Last observation carried forward (LOCF) is a recommended single-imputation method for dealing with missing data in TBI research clinical trials because it is conservative with respect to evaluation of the intervention.^5^ One recognized version of this approach is to substitute the 3-month outcome for missing 6-month data.^6,7^ Although LOCF is easy to understand and implement, the technique is suboptimal in several respects. First, it is biased in that it ignores potential time trends in GOS(e) trajectories. Second, application of LOCF is inefficient, because it neglects data observed briefly after the target time window. For example, a GOS(e) value recorded at 200 days post-injury is likely to be more informative about the status at 180 days post-injury than a value observed 90 days post-injury. Finally, the *ad hoc* nature of the LOCF method implies that there is no probabilistic model, and therefore no measure of uncertainty concerning the imputed values. This also implies that it is impossible to include additional covariates to further reduce bias introduced by the imputation method and that LOCF cannot be used to obtain multiply imputed data sets by design. Statistical Imputation of patient outcomes is gradually gaining acceptance in the TBI field as a method of dealing with missing data. Recent longitudinal studies have successfully employed techniques for both single^6,8,9^ and multiple imputation.^10–13^

Model-based imputation may not only be of value in cases of missing outcomes, but also for dealing with effects of broad time windows for assessments. The variation in timing of outcome assessments for patients with TBI varies among studies. Some studies define very stringent time windows (e.g. ±2 weeks; https://tracktbi.ucsf.edu/researchers), but in some contexts this can lead to a substantial amount of missing data.^3^ Consequently other studies have defined more pragmatic protocol windows (e.g., −1 month to +2 months^14^). Although the wider windows enable more complete data collection, they suffer from the problem that outcome can be evolving over this period, and an outcome assessment obtained at 5 months (the beginning of this window) in one subject may not be strictly comparable with outcomes obtained just before 8 months (the end of the window) in another subject. Consequently, even where outcomes are available within pragmatic protocol windows, there may be a benefit from being able to impute an outcome more precisely at the 180 day (6 month) time point.

In this article, four model-based imputation strategies for GOSe at 6 months ( = 180 days) post-injury in the longitudinal Collaborative European NeuroTrauma Effectiveness Research in Traumatic Brain Injury (CENTER-TBI) study^14^ are compared with LOCF with respect to their single-imputation performance. The focus on single-imputation is because the imputed values are to be integrated in the CENTER-TBI database and used in subsequent analyses by investigators. We examine four different model-based approaches – a panel imputation approach using multiple imputation via chained equation (MICE), a mixed-effects model (MM), a Gaussian process (GP) regression, and a multi-state model (MSM) – for imputing cross-sectional GOSe at 6 months exploiting the longitudinal GOSe measurements. Each model is fit in a version with and without baseline covariates.

## Methods

### Study population

The CENTER-TBI project methods and design are described in detail elsewhere.^14^ Participants with TBI were recruited into three strata: (1) patients attending the emergency room, (2) patients admitted to hospital but not intensive care, and (3) patients admitted to intensive care. Follow-up of participants was scheduled per protocol at 2 weeks, 3 months, and 6 months in group (1) and at 3 months, 6 months, and 12 months in groups (2) and (3). The protocol time window for the 6-month GOSe was between −1 and +2 months from the 6-month time point (5–8 months post-injury). Outcome assessments at all time points included the GOSe. The GOSe has the following categories: (1) dead, (2) vegetative state, (3) lower severe disability, (4) upper severe disability, (5) lower moderate disability, (6) upper moderate disability, (7) lower good recovery, and (8) upper good recovery. The GOSe was collected using structured interviews^15^ and patient/carer questionnaires.^16^ Since the latter do not identify vegetative patients as a separate category, the vegetative state and lower severe disability were combined in one group.

The study population for this empirical methods comparison were all individuals from the CENTER-TBI database (total of 4509) whose GOSe status was recorded at least once within the first 18 months and who were still alive 180 days post-injury (*n* = 3343). The rationale for conducting the comparison conditional on 6-month survival is simply that the GOSe can only be missing at 6 months if the individuals are still alive, because the GOSe score would be “dead” otherwise. Data for the CENTER-TBI study were collected through the Quesgen electronic-case report form (Quesgen Systems Inc, USA), hosted on the International Neuroinformatics Coordinating Facility (INCF) platform and extracted via the INCF Neurobot tool (https://neurobot.incf.org/). Release 1.1 of the database was used (see Supplementary Text for details). Basic summary statistics for population characteristics are listed in Table 1.

**Table 1.** Baseline Descriptive Variables Stratified by Applicability of LOCF

	*n*	No LOCF possible (*n* = 118)	LOCF possible (*n* = 3225)	*p*
Age	3343			0.46
Median (interquartile range)		47.0 (29.0–61.1)	49.0 (29.0–64.0)	
Range		7.0–90.0	0.0–95.0	
Sex: Male	3343	65/118 (55.085)	2145/3225 (66.512)	0.01
Stratum	3343			0.87
Emergency room		22/118 (18.644)	664/3225 (20.589)	
Admission to hospital		43/118 (36.441)	1155/3225 (35.814)	
Intensive care unit		53/118 (44.915)	1406/3225 (43.597)	
Cause of injury	3334			0.11
Road traffic incident		57/118 (48.305)	1250/3216 (38.868)	
Incidental fall		38/118 (32.203)	1440/3216 (44.776)	
Other		14/118 (11.864)	316/3216 (9.826)	
Violence/assault		7/118 (5.932)	146/3216 (4.540)	
Unknown		2/118 (1.695)	64/3216 (1.990)	
ISS, total	3305			0.39
Median (interquartile range)		16 (9–27)	16 (9–26)	
Range		1–75	1–75	
GCS	3236			0.62
Mild		82/115 (71.304)	2297/3121 (73.598)	
Moderate		8/115 (6.957)	252/3121 (8.074)	
Severe		25/115 (21.739)	572/3121 (18.327)	
Marshall CT	3030			0.87
1		49/106 (46.2264)	1206/2924 (41.2449)	
2		40/106 (37.7358)	1230/2924 (42.0657)	
3		3/106 (2.8302)	82/2924 (2.8044)	
4		0/106 (0.0000)	16/2924 (0.5472)	
5		0/106 (0.0000)	6/2924 (0.2052)	
6		14/106 (13.2075)	384/2924 (13.1327)	
Subarachnoid hematoma: yes	3262	41/115 (35.652)	1131/3147 (35.939)	0.95
Extradural hematoma: yes	3243	7/113 (6.1947)	356/3130 (11.3738)	0.09
Hypoxia: yes	3167	7/109 (6.4220)	170/3058 (5.5592)	0.70
Hypotension: yes	3193	6/110 (5.4545)	178/3083 (5.7736)	0.89
Glucose [mmol/L]	2548			0.90
Median (interquartile range)		6.8 (5.9–8.3)	6.9 (5.9–8.2)	
Range		3.7–15.7	1.9–33.5	
Hemoglobin [g/dL]	2802			0.67
Median (interquartile range)		13.6 (12.4–14.6)	13.5 (12.0–14.6)	
Range		8.1–17.1	1.3–23.4	

Last observation carried forward (LOCF) is not applicable when no Glasgow Outcome Sacle extended (GOSe) observation prior to 180 days is available*. n* is the number of non-missing values. *P* values are based on the χ^2^ test for binary variables and on the Wilcoxon test for continuous variables.

ISS, Injury Severity Score; GCS, Glasgow Coma Scale; CT, computed tomography.

**FIG. 1.**
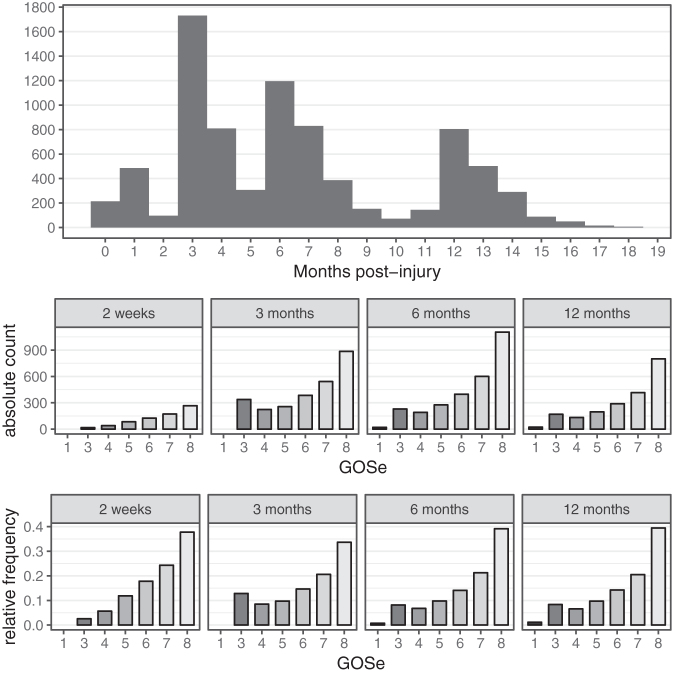
Glasgow Outcome Scale extended (GOSe) sampling time distribution and distribution at per-protocol time points (actual date rounded to nearest assessment window).

We decided to use only those GOSe observations obtained between injury and 18 months post-injury, because extremely late follow-ups were considered uninformative for the index follow-up time point of 6 months post-injury. This led to a total of 8185 GOSe observations of the study population being available for the analyses. For 1151 (34%) individuals, GOSe observations at 180 ± 14 days post- injury were available, and 2394 (72%) individuals had GOSe observations within the per-protocol window of 5–8 months post-injury. The distribution of GOSe sampling times and both absolute and relative frequencies of the respective GOSe categories are shown in Figure 1. True observation times were mapped to categories by rounding to the closest time point; for example, the “6 months” category contains observations up to 9 months post-injury. Thus, the figures include a small proportion of GOSe 1 representing patients who died between 6 and 9 months post-injury.

### Imputation methods

We compared LOCF to a MICE panel regression approach (multiple imputation [MI]), an MM, a GP regression, and an MSM. For all model-based approaches we additionally explored variants including the key IMPACT  = 2 predictors as covariates.^7^ These are age, Glasgow Coma Scale (GCS) motor score, pupil reactivity (0, 1, 2), hypoxia, hypotension, Marshall computed tomography (CT) classification, traumatic subarachnoid hemorrhage, epidural hematoma, glucose, and hemoglobin.

### LOCF

Because LOCF is widely used to impute missing outcomes in TBI studies,^6,7,9^ it served as the comparator method. Here, LOCF was defined as the last GOSe observation before the imputation time point of 180 days post-injury. LOCF is not a model-based method and, by definition, only permits the imputation of a GOSe value for subjects for whom at least one value was available within the first 180 days post- injury. We accounted for this lack of complete coverage under LOCF by performing all performance comparisons including LOCF only on the subset of individuals for whom a LOCF-imputed value could be obtained.

### Model-based methods

Model-based imputation approaches offer richer output (probabilistic imputation, multiple imputation) and may reduce the LOCF-inherent bias. We compared the performance of four model-based approaches with that of LOCF.

The MICE regression approach (MI) is a standard approach to MI that defines regression models for each missing variable in a matrix.^17^ By iterating over each variable that contains missing values, and resampling missing values from the corresponding regression model while holding all other variables fixed, a steady-state can ultimately be reached, and a set of imputed data sets can be generated. Because our goal is single imputation, we reduced the set of imputed values to a prediction by taking the most frequently imputed GOSe value. The frequency distribution of the imputed GOSe values can be used as probabilistic prediction in very much the same way as the probabilistic output of other model-based methods. To incorporate the longitudinal aspect of GOSe, we jointly imputed GOSe at 2 weeks, 3 months, 6 months, and 12 months jointly. This means that the GOSe at 2 weeks, 3 months, and 12 months act as covariates in the regression model for the 6-month GOSe.

MM are a widely used approach in longitudinal data analysis and model individual deviations from the population mean trajectory.^18^ The MM used for the GOSe imputation incorporates time as a non-linear covariate via a spline to be able to capture the non-linear dynamics of GOSe over time in the population. The MM was fitted using Bayesian methods to allow for the inclusion of patient-specific quadratic random effect (see Supplementary Text for details). An alternative non-linear regression model for longitudinal data is GP, which allows flexible modelling of both the individual GOSE trajectories and the population mean in a Bayesian non-parametric way.^19^ Both the employed MM and the GP are non-linear regression techniques for longitudinal data. Although these are powerful tools to model longitudinal trajectories, they do not explicitly model the probability of transitions between GOSe states. Because the number of observations per individual is limited in our data set (1–4 GOSe observations per individual), an approach explicitly modeling transition probabilities might be more suitable for capturing the dynamics of the GOSe trajectories. To explore this further, a Markov MSM was considered.^20,21^

All models were fitted using either none or all IMPACT predictors except for the MSM model, which only used age because of issues with numerical stability. Computational intensity is hard to compare because it depends on the exact hardware used. All methods except MSM can at least partially be run in parallel. On a Mac Book Pro 2019, the required time to fit each of the models on the entire available CENTER TBI data (with IMPACT covariates) was 13 min (MI), 26 min (MSM), 86 min (MM), and 112 min (GP). Although these differences are substantial, they are within one order of magnitude and would not preclude any of the methods in practice. Further details on the implementations are given in the Supplementary Text.

All models, irrespective of the fact whether they are Bayesian or frequentist, produce probabilistic outputs; that is, a discrete probability distribution over the possible GOSe values at 6 months for each individual. Although we propose only to store these probabilities along with the most likely GOSe value at 6 months, multiple imputations can be obtained post-hoc by resampling from the discrete probability distribution of each individual via inverse transform sampling.^22^ The functions required to sample from a discrete probability distribution are available in any statistical software package.

All four model-based approaches allow unbiased inference under a “missing at random” (MAR) mechanism^23^ Here, MAR means that whether or not a GOSe observation is missing is independent of the true functional outcome status of the individual. GOSe is an interview-based assessment that can also be completed by a proxy. The main operational challenge for consistent collection of longitudinal GOSe for observational studies therefore lies in the organization and scheduling of the interviews. A MAR assumption for CENTER data was therefore deemed plausible, albeit is not testable.^23^

### Performance assessment

Model performance was assessed via threefold cross-validation on the subset of individuals with a valid GOSe value within 180 ± 14 days post-injury (*n* = 1083). All models were fit on the entire available data after removing the 180 ± 14 days post-injury observation from the respective test fold, thus mimicking a missing completely at random missing data mechanism. The distribution of GOSe values in the three test sets was well balanced, (Fig. S1). All confusion matrices are reported as averages over the threefold cross-validation test sets. The column fraction confusion matrices are normalized within each category of observed GOSe value and are thus estimates of confusion, probability conditional on the observed GOSe. Performance was assessed using the absolute-count and the normalized (proportions) confusion matrices as well as bias, mean absolute error (MAE), and root mean squared error (RMSE). Bias is calculated by averaging the signed differences between observed and imputed values. A negative value of bias signifies that predicted values are lower overall than observed values, and a positive value means that they are higher. If differences cancel each other out, then bias can be zero even if the the predictions are inaccurate. MAE employs the unsigned differences, and it therefore gives a measure of accuracy irrespective of whether imputed outcomes are higher or lower than observed. In the calculation of RMSE the differences are squared, which penalizes large deviations from the target value more strongly than small ones. For example, the MAE will be 0.5 if 50% of imputed values agree with observed values and 50% differ by one category. The same MAE will arise if 75% agree exactly and 25% disagree by two categories. In the former case the RMSE will be 0.71 and in the latter case it will be 1.0.

These metrics have some limitations with ordinal data, and we therefore also considered directional bias (d-bias), which was calculated as the difference between the model-fitted probability of exceeding the observed value and the model-fitted probability of undershooting the observed GOSe as an alternative measure of bias. It is important to note that the scale of the directional bias is not directly comparable with the one of the other three quantities. All measures were calculated in the data set that was conditional on the ground truth (observed 6-months GOSe) as well as averaged over the entire test set.

LOCF, by design, cannot provide imputed values when there are no observations before 180 days post-injury. A valid comparison of LOCF with the other methods must therefore be based on the set of individuals for whom an LOCF imputation is possible. Overall, 118 out of 1083 test cases (10.9%) could not be imputed with the LOCF approach. In the entire study population, 345 individuals (10.3%) did not have data that would permit an LOCF imputation. The subset used for comparison of the imputation approaches with the LOCF approach was similar to the overall data set (Table 1).

## Results

**FIG. 2.**
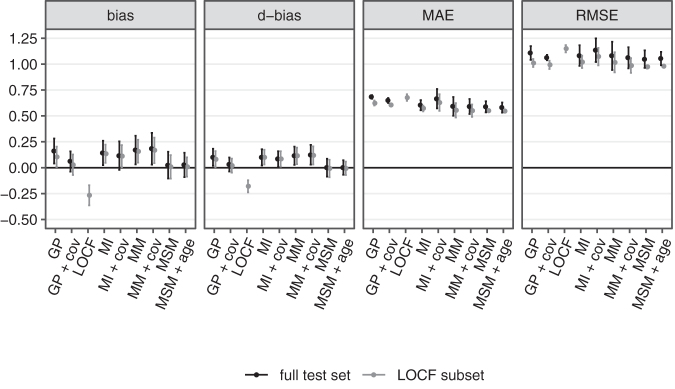
Cross-validated overall performance for all fitted models on the last observation carried forward (LOCF) subset (allowing LOCF) and the entire test set (LOCF performance not shown).

The overall performance of all fitted models in terms of bias, d-bias, MAE, and RMSE is depicted in Figure 2 both conditional on LOCF being applicable (gray) and, excluding LOCF, on the entire test set (black). Values are reported as mean over the three cross-validation folds and error bars indicate ±1.96 standard errors.

Several key findings are worth highlighting. First, LOCF is overall negatively biased; that is, on average it imputes lower-than-observed GOSe values. This reflects a population average trend toward continued recovery within the first 6 months post-injury. The fact that both ways of measuring bias qualitatively agree, suggests that application of these metrics is reasonable for the data. In terms of MAE and RMSE, LOCF also has worse performance, but differences among methods are less pronounced than for measures of bias. Notably, the RMSE difference between LOCF and the other methods is slightly larger than the MAE difference, which indicates that LOCF tends to produce more large deviations; that is, across several GOSe categories.

Second, including baseline covariates only produces clinically meaningful impact in the case of the GP regression model. The MI, MM, and MSM models perform more or less the same irrespective of adjustment for baseline covariates. This indicates that the additional predictive value of baseline covariates over the information contained in at least one observed GOSe value is limited. Further, both variants of the MI model and the MM fail to correct the overall bias of the imputed values.

We proceed with a detailed analysis of a subset of models both in direct comparison with LOCF and in the entire data set, including those cases in which LOCF is not applicable. In the following we only consider the baseline-adjusted GP model (“GP + cov”), the MI model without baseline covariates, the MM without baseline covariates, and the MSM without baseline covariates. The rationale behind dropping baseline adjustment for MI, MM, and MSM is that the additional complexity does not substantially alter overall performance. On the other hand, the GP model benefits from the inclusion of the IMPACT baseline covariates.

### Detailed comparison conditional on LOCF subset

**FIG. 3.**
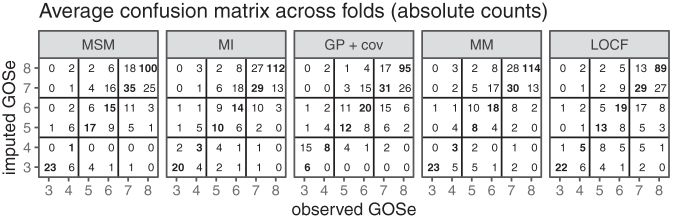
Confusion matrices averaged across folds (last observation carried forward [LOCF] subset only); absolute counts. Note that imputation is performed with the relevant observed values (test set) of the Glasgow Outcome Scale extended (GOSe) removed.

We first consider the results for the set of test cases that allow LOCF imputation (*n* = 965). Both the raw count and the relative (by left-out observed GOSe) confusion matrices are presented in Figure 3. The GOSe scale is restricted to 3+ since the imputation is conditional on an observed GOSe larger than 1 (deaths are known and no imputation necessary) and GOSe 2 was not distinguished as a separate category.

The absolute-count confusion matrices show that most imputed values are within (±) one GOSE category of the observed ones, and this yields an RMSE of ∼1. However, they also reflect the category imbalance (Fig. 1) in the study population. The performance conditional on the (in practice removed) observed GOSe value clearly shows that imputation for the most infrequent category 4 is the hardest. This is true across the range of methods considered. Both the MSM and the MM models account for this difficulty by almost never imputing a GOSe of 4. Instead, the respective cases tend to be imputed to GOSe 3 or 5.

**FIG. 4.**
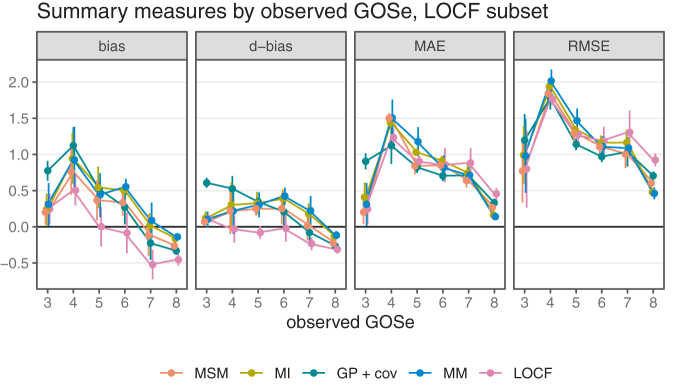
Performance measures by observed Glasgow Outcome Scale extended (GOSe); last observation carried forward (LOCF) subset only.

To better understand the overall performance assessment in Figure 2, we also consider the performance conditional on the respective ground truth (i.e., the observed GOSe categories in the test sets). The results are shown in Figure 4 (vertical bars are ±1.96 standard error of the mean).

Just as with overall performance, differences are most pronounced in terms of bias. Interestingly, the assessment conditional on LOCF being feasible reveals differences between bias as the difference between mean imputed and mean observed values and the difference in the probability of over- or undershooting the observed value. Again, the category imbalance in the GOSe distribution explains the fact that all model-based approaches tend to perform better for the most frequent categories (6, 7, and 8) while sacrificing performance for the less frequent categories (4 and 5) compared with LOCF. With respect to bias, all methods exhibit a certain regression to the mean effect, because low categories tend to be confused with better (higher) GOSe on average while high observed GOSe values are subject to a negative bias (at GOSe 7 and 8). Because LOCF does not take the category imbalance into account and because it exhibits a relatively large negative bias at the most frequent GOSe values, it is overall negatively biased. The conditional assessment of the GP regressions bias profile reveals overall unbiasedness, but this is the consequence of the relatively high positive and negative biases conditional on low/high GOSe values canceling each other out in the overall population. The MI, MSM, and MM models are fairly similar with respect to accuracy, but MSM clearly dominates with respect to bias. Note that irrespective of the exact definition of bias used, MSM dominates the other model-based approaches. Comparing LOCF and MSM, there is a slight advantage of MSM in terms of accuracy for the majority classes 3, 7, 8, which explains the overall difference shown in Figure 2. With respect to bias, MSM also performs better than LOCF for the most frequently observed categories, but the extent of this improvement depends on the performance measure.

### Detailed comparison on full test set

**FIG. 5.**
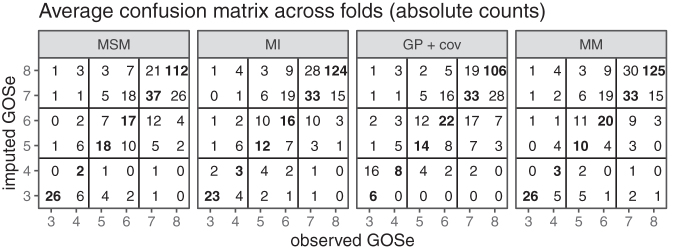
Confusion matrices averaged across folds (entire test set, no last observation carried forward [LOCF]); absolute counts. Note that imputation is performed with the relevant observed values (test set) of the Glasgow Outcome Scale extended (GOSe) removed.

**FIG. 6.**
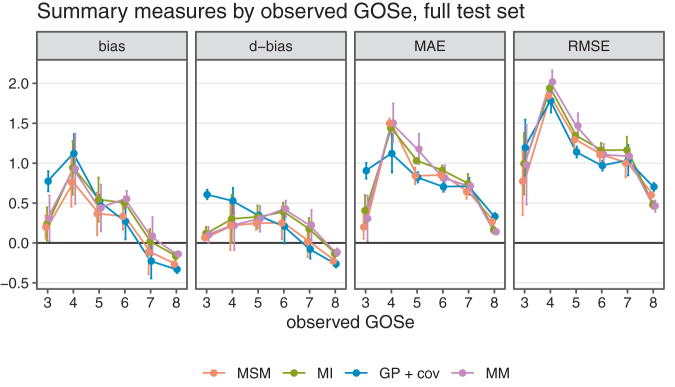
Performance measures by observed Glasgow Outcome Scale extended (GOSe); last observation carried forward (LOCF) subset only.

LOCF was not considered in the analysis of the full data set, because no LOCF was available for subjects with a first recorded outcome assessment >6 months post-TBI, and this renders a meaningful comparison across the entire data set impossible. The qualitative performance of the three remaining imputation approaches in the complete data set was similar to their performance in the subset of data used for comparison with LOCF (Figs. 5 and 6).

## Discussion

Handling missing data post-hoc to mitigate biases in analyses often requires great effort. It is therefore of the utmost importance to implement measures for avoiding missing data in the first place. Nevertheless, in practice, missing values resulting from loss-to-follow-up will always occur and should be addressed effectively.^3^ There is a wide consensus that statistically sound imputation of missing values is beneficial both for the reduction of bias and for increasing statistical power. The current gold standard for imputing missing values is multiple imputation on a per-analysis basis, including analysis-specific covariates to further reduce bias and to preserve imputation uncertainty in the downstream analysis. In practice, however, there are good reasons for providing a set of single-imputed default values in large observational studies such as CENTER-TBI. CENTER-TBI is committed to providing a curated database to facilitate multiple subsequent analyses. Because one of the primary end-points in CENTER-TBI is functional outcome at 6 months, a single default imputed value for as many study participants as possible is desirable. Consortia are increasingly committed to making their databases available to a range of researchers. In fact, more liberal data-sharing policies are becoming a core requirement for funding bodies (cf. https://www.openaire.eu/). In this context, it might not be possible to ensure that every analysis team has the necessary statistical expertise to properly conduct a per-analysis multiple imputation in the future. Further, the imputed values of a multiple-imputation procedure are inherently random, and it is therefore difficult to ensure consistency across different analysis teams if the values themselves cannot be stored directly in a database. For this reason, as a practical way forward, we suggest providing a default single-imputation together with a predictive distribution (value probabilities) for key outcomes in the published database itself. This mitigates problems with complete-case analyses and provides a principled and consistent default approach to handling missing values. Given the strong case for employing model-based approaches to imputation, it makes good sense to provide the predicted probabilities for each GOSe outcome in the core database alongside single imputed values as a transparent method for quantifying confidence in the imputation prediction. Based on these probabilities, it is easy to draw samples for a multiple imputation analysis if needed. Because we did not find any of the common predictors of GOSe to have a substantial effect on the imputed values in the presence of at least one observed GOSe value (at time point other than 6 months), the imputed values can be used in a wide range of subsequent analyses.

Wherever necessary and practical, a custom, analysis-specific multiple imputation approach might still be employed. In these cases, the model providing the single-imputed values may be used as a starting point.

Several reasons disqualify LOCF as method of choice. Not only is it inherently biased, but it is also inefficient in that it fails to properly account for the category imbalance of GOSe in the respective target population. Albeit simple to implement, LOCF – by definition – is not capable of exploiting longitudinal information obtained after the target time point. This results in a smaller subset of individuals for whom imputed values can be provided in the first place. LOCF also lacks the flexibility to adjust for further covariates, which might be necessary in some cases to further reduce bias under an MAR assumption. Finally, LOCF cannot produce an adequate measure of imputation uncertainty, as it is not model based.

Given this context, we draw two main conclusions from our comparison of three alternative, model-based approaches.

First and despite its theoretical drawbacks, LOCF is has the greatest accuracy (both MAE and RMSE ). The main advantages of a model-based approach are therefore, the ability to impute values for the entire study population, the reduction of bias, and the ability to provide a measure of uncertainty (value probabilities) together with the imputed values (or to use the same model to draw multiple imputations), as well as the possibility of including further analysis-specific covariates.

Second, we found that the inclusion of established baseline predictors had little effect on the imputation quality. It is important to note that this does not refute their predictive value, and the IMPACT covariates may be more relevant in studies confined to moderate and severe injuries. However, the current study suggests that there is little added benefit once at least one GOSe value is known. Differences among the various model-based approaches are rather nuanced. The more complex longitudinal models (GP, MM) that were fitted using Bayesian methods did not perform better, and because of the inherent complexities of a Bayesian analyses (e.g., convergence assessment) we discourage their use for the specific application at hand. The more standard MI-based approached achieved similar performance to the MSM approach in terms of precision (MAE or RMSE) but did not remove bias completely. We therefore favor the MSM for several reasons. It is easily interpretable in terms of transition intensities, and an efficient implementation is available^21^ in standard statistical software.^24^ Finally, it is the only method considered here that succeeds in eliminating the bias observed with LOCF. As with all other model-based imputation methods, MSM is able to provide imputed values for the entire population and to provide a probabilistic output to quantify imputation uncertainty.

## Supplementary Material

Supplemental data

Supplementary Text

## Research Strategy

CENTER-TBI is committed to reproducible research. To this end, the entire source code to run the analyses is publicly available at https://git.center-tbi.eu/kunzmann/gose-6mo-imputation. Scripts for automatically downloading the required data from the central access restricted “Neurobot” (https://neurobot.incf.org/) database at https://center-tbi.incf.org/ are provided. The analysis is completely automated using the workflow management tool “snakemake^25^” and a singularity^26^ container image containing all required dependencies is publicly available from zenodo.org (DOI: 10.5281/zenodo.2600385). Detailed step-by-step instructions on how to reproduce the analysis are provided in the README.md file of the GitLab repository.
