# Supplementary material for: Imputation of Ordinal Outcomes: A Comparison of Approaches in Traumatic Brain Injury
Source: J Neurotrauma. 2021 Jan 29;38(4):455–63. doi: 10.1089/neu.2019.6858 (PMC7875604; doi:10.1089/neu.2019.6858)
Supplement: Supplemental data [file Supp_FigS1-S2.zip › figures captions.docx]

*[file: KunzmannFig7.eps]*

Figure A.1: Marginal distribution of GOSe over the three cross validation folds.

*[file: KunzmannFig8.eps]*

Figure A.2: Structural diagram of allowed transitions between GOSe states for the proposed multi-state model.
