# Supplementary material for: Imputation of Ordinal Outcomes: A Comparison of Approaches in Traumatic Brain Injury
Source: J Neurotrauma. 2021 Jan 29;38(4):455–63. doi: 10.1089/neu.2019.6858 (PMC7875604; doi:10.1089/neu.2019.6858)
Supplement: Supplementary Text [file Supp_Data.pdf]

Supplementary Text

Ethical Approval Statement

The CENTER-TBI study (EC grant 602150) has been conducted in accordance with all relevant laws of the EU if directly applicable or of direct effect and all relevant laws of the country where the Recruiting sites were located, including but not limited to, the relevant privacy and data protection laws and regulations (the ''Privacy Law''), the relevant laws and regulations on the use of human materials, and all relevant guidance relating to clinical studies from time to time in force including, but not limited to, the ICH Harmonised Tripartite Guideline for Good Clinical Practice (CPMP/ICH/135/95) (''ICH GCP'') and the World Medical Association Declaration of Helsinki entitled ''Ethical Principles for Medical Research Involving Human Subjects''. Informed Consent by the patients and/or the legal representative/next of kin was obtained, accordingly to the local legislation, for all patients recruited in the Core Dataset of CENTER-TBI and documented in the e-CRF. Ethical approval was obtained for each recruiting site. The list of sites, Ethical Committees, approval numbers and approval dates can be found on the website: https://www.center-tbi.eu/project/ethical-approval.

Distribution of GOSe in Validation Folds

Models

MI approach. Multiple imputation via chained equations (MICE) is a standard approach to multiple imputation.[17] Instead of specifying a full (longitudinal) model for GOSe the MI approach uses a so called ''fully conditional approach'' to specify a model for each variable in a dataset given all other variables. By sequentially refitting these conditional models and then resampling missing values a set of imputed datasets can be generated. For the GOSe prediction, we treated the GOSe at the nominal query points of two weeks, 3 months, 6 months, and 12 months as separate variables and imputed them using the mice package for the R programming language.[24] We used proportional odds ordinal regression [27] as conditional models for the ordinal outcome GOSe at the four different time points and ran the algorithm with 100 chains up to convergence. This means that 100 potential GOSe outcomes per individual with missing GOSe at 6 months are available upon convergence of the procedure. The final probabilistic predictions where then taken as the relative frequencies of the imputed outcomes and the point prediction as the most frequently imputed value.

Mixed-effects model. Mixed effects models are a widely used approach in longitudinal data analysis and model individual deviations from a population mean trajectory.[18] To account for the fact that the GOSe outcome is an ordered factor, we employ a cumulative link function model with flexible intercepts.[27] The population mean is modeled as a cubic spline function to allow a non-linear population mean trajectory. Patient-individual deviations from this population mean are modeled as quadratic polynomials to allow sufficient flexibility (random effects). Baseline covariates are added as linear fixed effects to the population mean. The model was fitted using Bayesian statistics via the BRMS package[28,29] for the R environment for statistical computing[24] and the Stan modelling language for Markov Chain Monte Carlo sampling.[30] The required burn-in length to reach a steady state for the Markov Chains was determined by inspecting the trace plot of the model on the complete data set. During the cross validation fits the same burn-in length was used and convergence was assessed via the potential scale reduction factor (PSRF) proposed by Gelman and Rubin.[31] A Bayesian approach was necessary since the quadratic random effect is not identifiable in individuals where only one or two GOSe values are available. The model with cubic spline fixed effect for time and quadratic random effects for time per individual was selected based on the highest expected log predictive density on the full data set (computed via the loo package[32], data not shown). Non-informative priors were used for the model parameters. These are sufficient to make all model parameter identifiable and effectively shrink the quadratic random effect in individuals with only two observed GOSe values to zero. A potential drawback of the proposed longitudinal mixed effects model is the fact that the individual deviations from the population mean are modeled globally using polynomials. Since linear and quadratic terms are not identifiable for patients with only one observed GOSE value, this implies large uncertainty over the patient-specific effects for individuals with one or two observations only by falling back on the non-informative priors on these model parameters. Thus, the overall uncertainty associated with model-based imputations at exactly 180 days may become relatively large for these individuals. A more flexible regression model might avoid this particular pathology which is why we also implement a Gaussian process regression model (see below).

Gaussian process model. Gaussian process regression allows flexible modelling of both the individual GOSE trajectories as well as the population mean in a Bayesian non-parametric way.[19] This non-parametric paradigm leads to low model-uncertainty in the vicinity of actually observed GOSe outcomes. To account for the discreteness of the GOSe outcome, the continuous output of the Gaussian process model is rounded to the nearest integer in 1 to 8 (GOSe categories). The squared exponential covariance function with shared length scale for all individuals is used to model intra-individual dependency of GOSe outcomes. The population mean trajectory of the Gaussian process is modeled as mean of an independent Gaussian process with pseudo observations at 45, 90, 180, 270, 360 days post-injury (also using a squared exponential covariance function). This approach maintains flexibility of the population mean function similar to the spline-based approach for the mixed effects model while avoiding the computational complexity of a fully hierarchical Gaussian process model. Again, the impact of baseline covariates is modeled via linear effects on the population mean of the Gaussian process. All parameters are estimated in a fully Bayesian fashion using the Stan modelling language[30] and non-informative priors except for the length scale of the squared exponential kernel. Due to the sparseness of the data, the estimated length scale will naturally tend towards extremely large values implying unrealistically long-range dependency

between observations. We therefore limit the length scale to a maximum of 120 days (4 months) and impose a Gaussian prior with a mean of 60 days post injury and a standard deviation of 14.

Multi-state model. Both the mixed effects model as well as the Gaussian process regression model are essentially non-linear regression techniques for longitudinal data. While they are both powerful tools to model longitudinal trajectories, they do not explicitly model the probability of transitions between GOSe states. Since the number of observations per individual is limited in our data set (1 to 4 GOSe observations per individual), an approach explicitly modelling transition probabilities might be more suitable to capture the dynamics of the GOSe trajectories. To explore this further, a Markov multi-state model is considered.[20] This model class assumes that the transitions between adjacent GOSe states can be modeled as a Markov process and the transition intensities between adjacent states are fitted to the observed data.

To account for the fact that state-transitions might be more frequent in the early post-injury phase, piecewise constant transition intensities were fitted to the intervals [0, 90), [90, 270), and 270+ days post-injury. The model was fit using the msm package[21] for the R environment for statistical computing.[24] Due to the relatively large number of 19 transition intensities in the proposed model (cf. arrows in Figure A.1, structure of transition graph), inclusion of all baseline covariates turned out to be numerically unstable. For the MSM model, instead of including all covariates, only a model adjusting for age at injury via a proportional hazard approach was fit.
